# Organic Field-Effect Transistors Based on a Liquid-Crystalline Polymeric Semiconductor using SU-8 Gate Dielectrics on Flexible Substrates

**DOI:** 10.3390/ma7117226

**Published:** 2014-10-29

**Authors:** Kornelius Tetzner, Indranil R. Bose, Karlheinz Bock

**Affiliations:** 1 Research Center for Microperipheric Technologies, Technische Universität Berlin, Gustav-Meyer-Allee 25, 13355 Berlin, Germany; 2 Fraunhofer Research Institution for Modular Solid State Technologies EMFT, Hansastr. 27d, 80686 Munich, Germany; E-Mail: indranil.bose@emft.fraunhofer.de; 3 Electronics Packaging Lab (IAVT), Technische Universität Dresden, Helmholtzstr. 10, 01069 Dresden, Germany; E-Mail: karlheinz.bock@tu-dresden.de

**Keywords:** liquid-crystal polymers, organic field-effect transistors, organic semiconductor, long-term stability

## Abstract

In this work, the insulating properties of poly(4-vinylphenol) (PVP) and SU-8 (MicroChem, Westborough, MA, USA) dielectrics are analyzed and compared with each other. We further investigate the performance behavior of organic field-effect transistors based on a semiconducting liquid-crystal polymer (LCP) using both dielectric materials and evaluate the results regarding the processability. Due to the lower process temperature needed for the SU-8 deposition, the realization of organic transistors on flexible substrates is demonstrated showing comparable charge carrier mobilities to devices using PVP on glass. In addition, a µ-dispensing procedure of the LCP on SU-8 is presented, improving the switching behavior of the organic transistors, and the promising stability data of the SU-8/LCP stack are verified after storing the structures for 60 days in ambient air showing negligible irreversible degradation of the organic semiconductor.

## 1. Introduction

The solution-based deposition of polymeric gate dielectrics in organic field-effect transistors (OFET) has become a very attractive way to realize low-cost and flexible organic electronic devices on large areas for low-cost applications by using several kinds of deposition techniques, such as spray-coating [1], printing [2] or even web-coating [3]. The basic requirements of a dielectric typically cover good electrical insulation to increase the on/off-current ratio of a transistor and to reduce power consumption and high capacitances in order to lower the supply voltages. In the case of bottom-gate devices, several additional demands become obvious, for instance good chemical resistance and wettability, to allow subsequent solution processes or low surface roughness to minimize the charge carrier trap density at the interface to the semiconductor, which drastically limits the choice of material [4,5,6,7]. To date, one of the most heavily used organic dielectric material is poly(4-vinylphenol) (PVP), due to its good insulating characteristic and excellent film-forming properties with smooth surfaces enabling the fabrication of OFET devices with mobilities up to 3 cm^2^/Vs [8]. However, this material also has several drawbacks, most of all the high cross-link temperatures at around 200 °C, in order to allow subsequent solution-based processes after the dielectric deposition, avoiding damage to the PVP films. Such high temperatures are unacceptable when using low-temperature plastic substrates, like polyethylene terephthalate (PET) or polyethylene naphthalate (PEN). Several alternative approaches have been presented recently to reduce the cross-link temperature, e.g., by using photoacid generators in combination with UV-light [9] or changing the cross-link agent [10], but often additional catalysts are required to ensure solubility stability [11] or the films do not maintain the electrical properties compared to thermally cross-linked PVP, lowering transistor performance [12]. Furthermore, it has been stated that OFETs are more susceptible to hysteresis issues when using PVP dielectrics, especially when being operated in ambient air, which is mainly caused by the internal hydroxyl groups [13,14].

A material that meets most of the demands for a dielectric in an OFET bottom-gate architecture was seen in this work in the negative photoresist, SU-8 (MicroChem, Westborough, MA, USA). This material can be cross-linked by a combination of low temperatures at around 100 °C and UV-light, with cross-linked films showing high resistance against a wide range of chemicals. The cross-link procedure also allows an easy patterning of the dielectric, which is essential for the realization of integrated circuits. SU-8 exhibits very high optical transparency above 360 nm, making it suitable for opto-electrical applications [15]. In addition, it has been stated as fully biocompatible, allowing the possibility to realize bio-MEMS and sensors [16]. SU-8 is an out-of-the-box material, which means that it can be processed as received without additional modification or mixing steps. Batch-to-batch variations are typically low, due to the high quality control of the manufacturer. The electrical characteristics are also very promising, with leakage current densities below 10−9 A/cm^2^, a dielectric strength of 4.5 MV/cm and a dielectric constant of three [17,18].

Up to now, less work has been done in the investigation of SU-8 as the dielectric for OFET devices, especially with solution-based deposition processes of the semiconductor on flexible substrates. Experiments with solution-deposited poly(3-hexylthiophen-2,5-diyl) showed weak performances of the OFET devices, probably due to the stability issues of the semiconductor [19] or interface-related issues between the semiconductor and dielectric [20]. Further work with evaporated pentacene as the active layer on glass substrates revealed the potential of SU-8 as a reliable dielectric material for OFETs with mobilities of 0.56 cm^2^/Vs, on/off-ratios up to 107 and good environmental stability of the devices for several days [21]. However, high driving voltages are needed for the transistors, mainly due to the high thicknesses of the dielectric (∼1 µm) and the low dielectric constant. Recent results from MicroChem demonstrated promising performances with semiconducting materials based on a blend of 6,13-bis(triisopropylsilylethynyl)pentacene (TIPS-PEN) and polytriarylamine from solution. The devices with a dielectric thicknesses of 350 nm exhibited on/off-ratios of 104 and threshold voltages of −1.18 V, but also large hysteresis [22]. However, these results were only achieved on rigid glass substrates.

In this work, we present the realization of OFET devices based on SU-8 dielectrics and a liquid-crystal polymer (LCP) semiconductor on flexible substrates using a foil-on-carrier (FOC) technology. The FOC technology has the advantage that highest versatility is achieved due to the fact that processes coming from silicon technology can be combined with processes used in organic electronics, like printing or dispensing. This technology has already proven to be an effective way for realizing highly flexible plastic foils containing complex integrated circuits [23]. We will show that the device performances achieved on such structures are comparable to those achieved on rigid substrates based on PVP dielectrics with high reproducibility and promising long-term stability. Furthermore, an automated dispensing process of the LCP on SU-8 dielectrics is introduced, demonstrating the feasibility of integrating this technology in a roll-to-roll mass production.

## 2. Experimental Section

### 2.1. Preparation of Substrates

Heat-stabilized PEN foils (Teonex Q831A) were kindly provided by DuPont Teijin Films with a thickness of 25 µm. This foil is purpose-made for lamination processes, since it features a temporary adhesive on one side. The foils were laminated on 4-inch silicon wafers exhibiting a 100-nm thermal silicon dioxide layer by using a manual laminator for dry film photoresists (Bungard RLM 419p, Windeck, Germany) at a temperature of 90 °C, a pressure of 8 bar and a speed of 0.5 m/min. After the lamination, the FOCs were put into a convection oven ramped up to 200 °C for 45 min to activate the adhesive and were taken out after 1 min at 200 °C to cool down to room temperature. Delamination after full processing is achieved by peeling off the foil from the wafer at elevated temperatures at 60 °C using minor force.

The FOCs were cleaned by softly wiping the surface with a cleanroom tissue soaked in acetone to remove larger particles followed by an ultrasonic bath in a detergent solution (Micro90 2%) and, subsequently, in deionized water for 10 min, respectively. Drying was carried out in a nitrogen-purged spin dryer. In order to fabricate OFET devices on a smooth surface, the FOC was additionally planarized by spin-coating a 5 µm SU-8 (SU-8 2, MicroChem) layer. Prior to SU-8 deposition, the FOCs were treated with an oxygen plasma (250 W, 2 min) and spin-cleaned with acetone and isopropyl alcohol to improve the wetting conditions. SU-8 was dispensed statically on the FOC and spin-coated at 1,000 rpm for 30 s. The as-spun film was soft-baked at 65 °C for 3 min followed by 95 °C for 5 min and then cooled

down to room temperature. To ensure a high cross-link reaction of the epoxy resin molecules, the SU-8 was overexposed using a MA150 Mask Aligner (SÜSS MicroTec, Garching, Germany) with an energy dose of 400 mJ/cm^2^. A final post exposure bake at 65 °C for 3 min and 95 °C for 5 min completed the polymerization between the resin molecules.

### 2.2. Fabrication of Organic Transistors

Bottom-gate, top-contact organic transistors were fabricated by sputtering a 100-nm layer of aluminum on the planarized FOC, which was patterned by using photolithography processes, forming the gate electrodes. In order to achieve a dielectric thicknesses below 1 µm, the SU-8 formulation had to be diluted in a volume ratio of 1:1 with the solvent, gamma-butyrolactone, and the spinning speed was set to 3000 rpm for 30 s. All other preparation steps of the SU-8 dielectric were carried out in the exact same way as was done for planarization. The LCP semiconductor FS111 (Flexink Ltd., Southampton, UK) was dissolved in p-xylene at a concentration ratio of 10 mg/mL and filtered through a 0.2-µm filter. The solution was spin-coated at 2000 rpm for 20 s onto the FOC substrates, followed by a drying step of the films on a hotplate at 100 °C for 5 min. Subsequently, gold source/drain electrodes were formed by evaporation through a shadow mask, resulting in thicknesses of 100 nm.

Reference transistor structures based on PVP dielectrics were fabricated on 4-in borosilicate glass wafers using the same processes employed for SU-8-based transistor devices on FOC. PVP (Sigma-Aldrich, St. Louis, MO, USA, M_w_∼25.000) was dissolved in n-butanol, and poly(melamine-co-formaldehyde) was added as the crosslinking agent at a concentration of 15% and 3%, respectively. The solution was filtered through a 0.44-µm syringe filter and spin-coated at 1500 rpm for 30 s onto the glass substrates exhibiting aluminum gate structures. The PVP dielectric film was baked for 5 min at 100 °C and finally cross-linked at 200 °C for 20 min followed by the semiconductor and top electrode deposition.

For comparative studies on the performance of the semiconductor, FS111, additional experiments on heavily doped p-type silicon substrates (Silicon Materials, Kaufering, Germany) with a 1000 µm thermally grown silicon dioxide (SiO_2_) dielectric were carried out.

All transistors used in this study exhibited channel widths of 3.5 mm and channel lengths of 100 µm.

### 2.3. Automated µ-Dispensing of the Semiconductor

In addition to spin-coating experiments, the feasibility of an additive deposition technique of the organic semiconductor, FS111, on the SU-8 coated FOC was evaluated using an automated µ-dispenser. The dispensed volume of the automated dispensing tool (Vermes dosing valve MDS 3020A, Otterfing, Germany) used in the experiments was adjusted, such that the droplets fully covered the gate electrodes to ensure the good functionality of the transistors. In addition, the semiconductor solution was thinned down from a concentration of 10 mg/mL to 2 mg/mL to further reduce the thickness of the semiconductor during the drying process. After the µ-dispensing, the substrates were baked on a hotplate at 100 °C, and gold source/drain electrodes were formed by evaporation through a shadow mask.

### 2.4. Characterization

Atomic force microscope (AFM) images were obtained in non-contact mode using an SIS ULTRAObjective (Fries Research & Technology, Bergisch Gladbach, Germany) with scanning speeds of 15 µm/s. Film thicknesses were measured using a Sentech SE 400adv ellipsometer (Sentech Instruments, Berlin, Germany) or a Dektak 200-Si profilometer (Bruker Corporation, Billerica, MA, USA). Electrical characterization of the capacitors and organic transistors was done with a Keithley 4200 semiconductor parameter analyzer (Keithley Instruments, Solon, OH, USA) for I-V measurements and an Agilent 4284A LCR meter (Agilent Technologies, Santa Clara, CA, USA) for the capacitance determination at a frequency of 1 kHz. The field-effect mobility µsat was calculated in the saturation regime from the linear fit of I_D_^1/2^ *versus* V_G_, and threshold voltage V_T_ was extrapolated from the intercept point of the linear fit and the V_G_-axis. The subthreshold swing was calculated by taking the inverse of the slope |I_Dsat_| *versus* V_G_ in the subthreshold regime.

We highlight that all fabrication and characterization steps were carried out in ambient air.

## 3. Results and Discussion

### 3.1. Surface Properties of SU-8 Planarization Films

**Figure 1:**
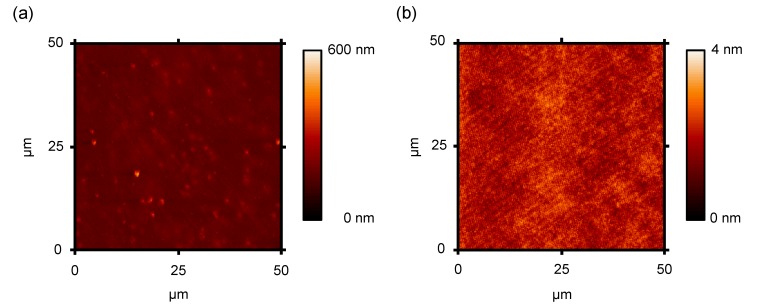
(**a**) AFM pictures of cleaned polyethylene naphtalate (PEN) and (**b**) SU-8 planarized PEN substrates using foil-on-carrier (FOC) technology.

The surface quality of SU-8 films was evaluated by AFM measurements on the FOC substrates before and after planarization on an area of 50 µm × 50 µm. The images of the measurements are shown in Figure 1. Several contaminants, such as particles and scratches, are visible on the pure PEN foil, which may arise from the facts that the foil was fabricated under non-cleanroom conditions and packed onto rolls, causing rubbing. Such defects could not be removed, even with intensive and extended washing. Calculations of the roughness parameters from the AFM measurement revealed a root mean square roughness R_q_ of 15 nm, and an average distance between the highest peak and the lowest valley of the profile R_z_ of 214 nm was determined. In contrast to this, the SU-8-coated foils showed very smooth surfaces, which clarifies that SU-8 can act as an excellent planarization layer covering all imperfections. R_q_ and R_z_ were this time in the range of 0.3 nm and 2.5 nm, respectively. These values are comparable to the roughness data given from commercial suppliers for borosilicate glass wafers. Subsequent layers of SU-8 acting as the dielectric showed similar results, which is beneficial for the formation of a defect-free interface between the dielectric and organic semiconductor.

### 3.2. Electrical Characterization of Organic Transistors Based on SU-8 and PVP Dielectrics

**Figure 2:**
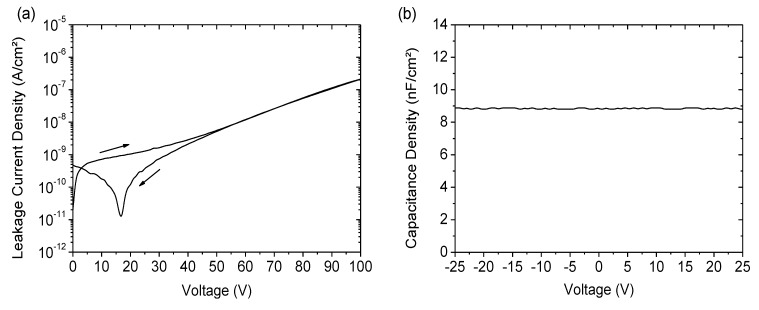
(**a**) Leakage current density and (**b**) capacitance density measurement for SU-8 dielectric films with thicknesses of 305 nm.

**Figure 3:**
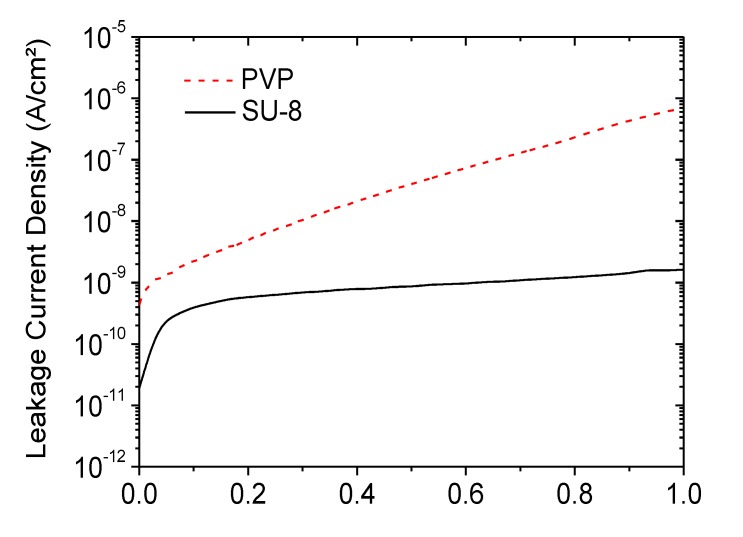
Comparison of leakage current densities for PVP and SU-8 dielectrics.

Ellipsometry measurements of SU-8 dielectrics on planarized FOC and PVP dielectrics on glass revealed thicknesses of 305 nm and 1.3 µm, respectively. Attempts to decrease the thickness of the PVP failed due to the formation of small pinholes in the layer caused by the high curing temperature. The final cross-linked PVP, as well as the SU-8 showed high chemical resistance against a wide range of solvents, including acetone, toluene, xylene and dichlorobenzene. Electrical characterization of the dielectrics was carried out by measuring the leakage current and the capacitance of the capacitor structures (Al/dielectric/Au). The results for SU-8 are plotted in Figure 2. The films showed good insulating properties, even at higher voltages, and excellent stability against high electrical fields is observed with the breakdown of the layers, typically occurring at 150 V, which equals a dielectric strength of 5 MV/cm. The extraction of the dielectric constant yielded a value of three, which is consistent with the specifications given in the literature. The SU-8 layer showed no signs of pinholes, and capacitor structures with areas of up to 20 mm^2^ had full functionality. In order to make a direct comparison of PVP and SU-8 dielectric layers exhibiting different thicknesses, the leakage currents for both materials as a function of the applied electrical field are plotted in Figure 3. It can be seen that the SU-8 dielectric shows a better insulation behavior, and the leakage currents are more than one order of magnitude higher for PVP dielectrics at the same electrical fields. In addition, the breakdown of the PVP already occurred at ∼1.5 MV/cm. Previous studies on the leakage current of PVP films with thicknesses comparable to our SU-8 layers revealed similar results, with values being still more than one order of magnitude higher [8,10]. Regarding our process and the environmental conditions during thin-film fabrication of the dielectrics, it is possible to state that SU-8 is not only better in terms of processability on flexible substrates, but also shows superior electrical properties compared to PVP films. This observation is supported by previous investigations on both materials as dielectrics for OFETs on rigid substrates and is probably due to the lower hygroscopicity of SU-8, preventing charge carrier injection through the dielectric [24]. The dielectric constant of PVP was determined to have a value of five.

**Figure 4:**
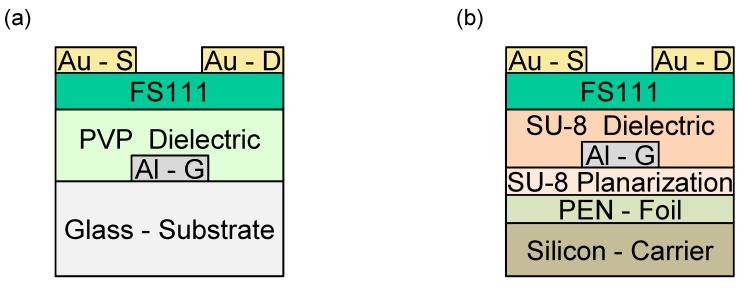
Device architectures for OFETs based on (**a**) PVP and (**b**) SU-8 dielectrics.

The device setup of organic transistors with PVP on glass wafers and SU-8 on FOCs is depicted in Figure 4. Measurement of the devices using the semiconductor, FS111, was carried out immediately after fabrication in the dark. FS111 is a proprietary donor-acceptor co-polymer exhibiting a HOMO level of 5.2 eV and has a number average molecular mass (M_n_) of 39.700 g/mol and a polydispersity of 2.53, as measured by gel permeation in chlorobenzene at 80 °C against polystyrene standards. Wide-angle X-ray diffraction measurements, as shown in Figure S1, have proven the formation of a liquid-crystalline mesophase, indicating a lamellar-like packing of the liquid-crystalline backbones with d-spacing of Å after annealing [25]. The small length scale of self-organization or crystallization compared to highly crystalline materials, like TIPS-PEN, facilitates the reproducibility over the length scale of the transistor channel. Promising results have been already achieved with preceding versions of this LCP material, showing comparable performances to TIPS-PEN with high device-to-device reproducibility [26]. The thickness of FS111 was approximately 60 nm for devices based on PVP and SU-8, as measured by ellipsometry.

**Figure 5:**
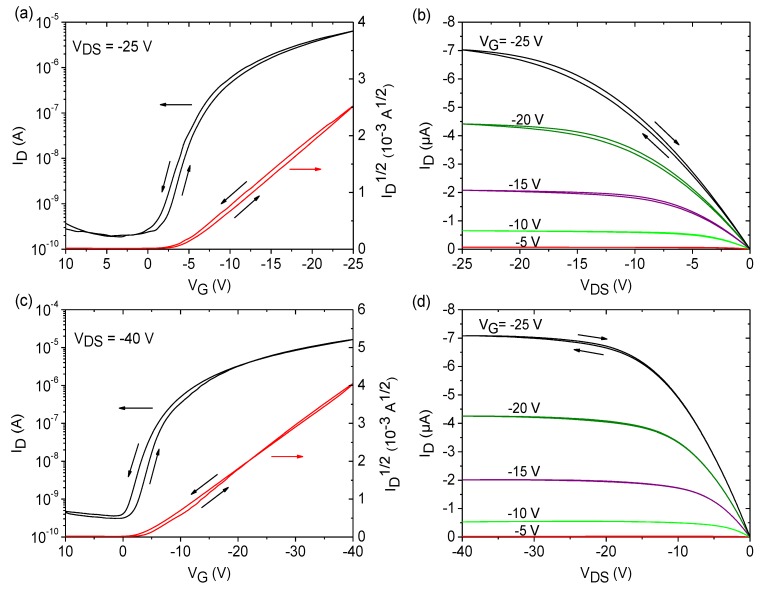
Representative transfer and output curves for OFET devices with the semiconductor, FS111, using SU-8 dielectrics on FOC at a supply voltage of −25 V (**a**,**b**) and PVP dielectrics on glass at a supply voltage of −40 V (**c**,**d**) with a channel width and length of 3.5 mm and 100 µm, respectively.

**Figure 6:**
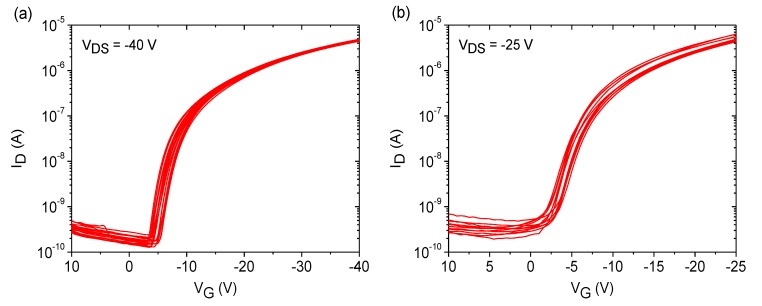
Set of a minimum of 12 transfer curves of OFET devices based on (**a**) PVP and (**b**) SU-8 dielectrics using FS111 measured on three test wafers for each dielectric material.

Measurement of the transistor devices was carried out directly after fabrication. Representative transistor curves for SU-8 and PVP dielectrics are depicted in Figure 5, and extracted transistor parameters are given in Table 1. From the characteristics and the parameters, it can be seen that the performance of both transistor devices is very similar. Negligible hysteresis effects are observed when using SU-8 and PVP dielectrics, and the variation of the transistor parameters is very low, demonstrating a good device-to-device reproducibility by the use of FS111. Figure 6 emphasizes the high uniformity of the OFET devices on PVP and SU-8 dielectrics in combination with the LCP semiconductor in which sets of a minimum of 12 transfer curves measured on three different wafers for each dielectric material is shown, proving the low spread in performance. Despite the fact the there were encouraging results for both dielectric materials, additional positive aspects become obvious regarding transistors based on SU-8 dielectrics. The use of this material allowed the realization of very thin dielectric layers, which, in turn, reduced the supply voltage down to −25 V. It should be noted that the parameters given in Table 1 for transistors using SU-8 and PVP were extracted at different supply voltages of −25 V and −40 V, respectively. Reduction of the supply voltages of PVP-based OFET devices down to −25 V revealed a degraded switching behavior of the transistor with a lower on/off-current ratio of 5 × 10^3^ and a higher subthreshold swing of 2.6 V/dec, as shown in Figure S2 and Table S1.

**Table 1:** Extracted parameters for FS111 using SU-8 dielectrics on FOC and PVP dielectrics on glass.

Characteristic	FS111 on SU-8/FOC	FS111 on PVP/glass
Field-effect mobility µ_sat_	0.1 cm^2^/Vs *±* 0.04 cm^2^/Vs	0.2 cm^2^/Vs *±* 0.03 cm^2^/Vs
Threshold voltage V_T_	−4 V *±* 0.8 V	−5 V *±* 1 V
On/off-current ratio	2 × 10^4^	3 × 10^4^
Subthreshold swing	1.6 V/dec	1.7 V/dec

**Figure 7:**
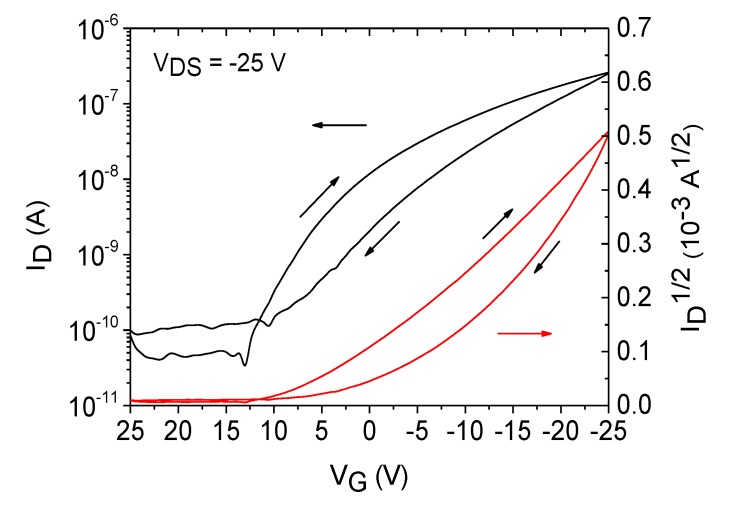
Transfer curve of an organic transistor based on FS111 on SiO_2_ dielectrics.

Furthermore, due to the better insulation properties of SU-8, gate leakage currents are approximately one order of magnitude lower compared to the leakage currents occurring in PVP-based devices. As depicted in Figure S3a,b, the current ratio ID/IG in the on state was approximately 10^2^ and 10^3^ using PVP and SU-8 dielectrics, respectively. The power consumption is therefore reduced when using SU-8 dielectrics. It should be mentioned that the active area of spin-coated FS111 was not patterned before the measurement, which leads to increased leakage currents showing higher I_D_ in the off state and higher I_G_ in the on state. Finally, the complete fabrication of organic transistors on foil based on SU-8 dielectrics was carried out at low temperatures with maximum values of 100 °C. In parallel to the experiments on organic dielectrics, additional investigations on the semiconductor performance was carried out on SiO_2_ dielectrics. The measurement results are presented in Figure 7. As can be seen, the devices show increased hysteresis effects, and the extracted field-effect mobilities were in the range of 0.006 cm^2^/Vs. We suggest a higher trap site density between the inorganic dielectric and the organic semiconductor, which causes a decrease in the performance of the organic transistor, as has been observed in previous investigations using SiO_2_ as the dielectric [5,27].

### 3.3. Automated µ-Dispensed Organic Transistors on Flexible Substrates

**Figure 8:**
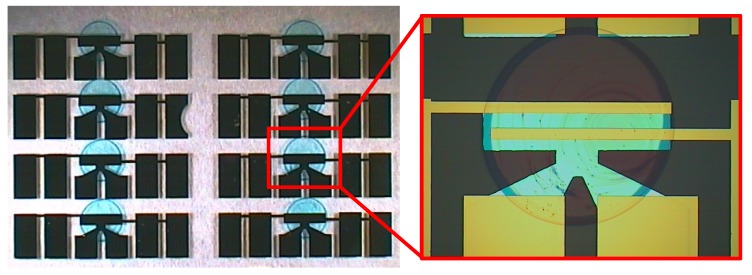
Set of eight transistors with drop-coated FS111 using an automated dispensing system and a single transistor showing the coverage of the semiconductor on the gate.

**Figure 9:**
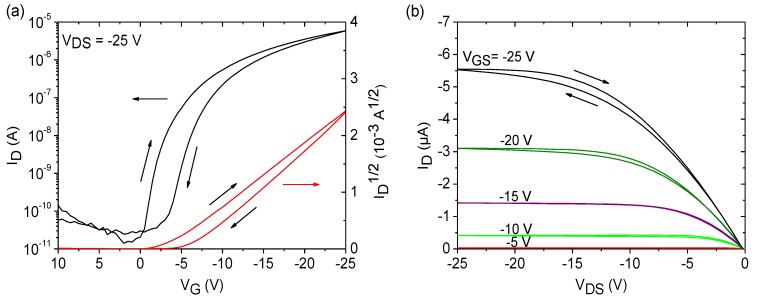
(**a**) Transfer and (**b**) output curve of organic transistors based on automated dispensing of FS111 on SU-8 dielectrics on PEN foil.

In order to structure a defined area of the organic semiconductor, an additive deposition technique of the LCP FS111 by µ-dispensing was employed using an automated dispensing system. The advantages of this technique are the reduction of material waste, improvement of the on/off-current ratios and the compatibility with large-area processing. Furthermore, it allows the fabrication of complex integrated circuits in which different kinds of organic semiconductors (e.g., n- and p-type) have to be deposited on one substrate from solution. The µ-dispenser allowed the deposition of a defined volume of the semiconductor in the nanoliter range, resulting in reproducible droplets with diameter variations of less than 5%. A set of several drop-coated transistors with uniform dispensed FS111 on planarized FOC with SU-8 as the dielectric is shown in Figure 8, and representative transistor curves are given in Figure 9.

**Figure 10:**
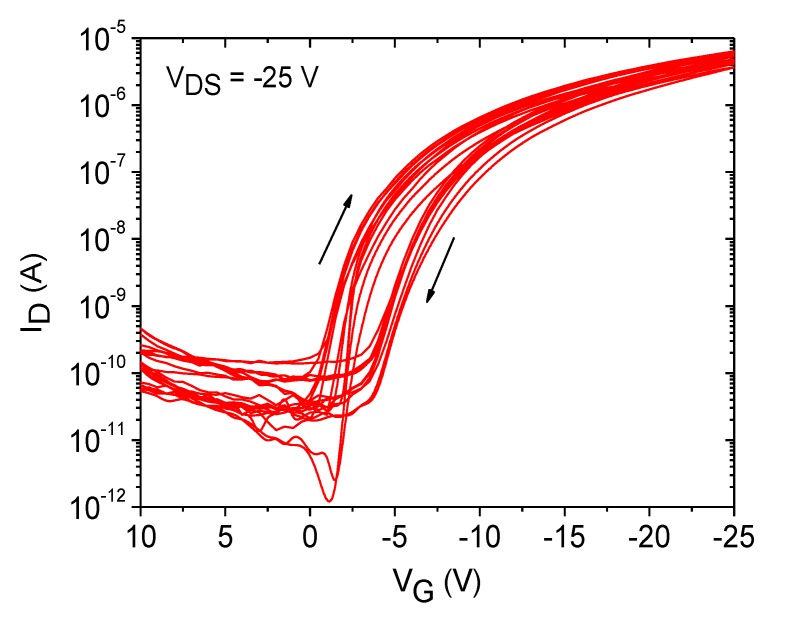
Set of 12 transfer curves of OFET devices on SU-8 dielectrics using an automated µ-dispensing process of the organic semiconductor, FS111.

**Figure 11:**
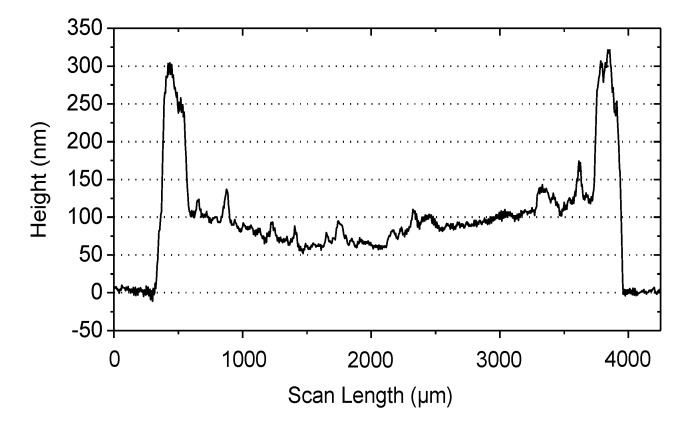
Profile scan of the µ-dispensed semiconductor, FS111, across the center of a droplet.

Extraction of the field-effect mobility, threshold voltage, subthreshold slope and on/off-current ratio yielded mean values of 0.09 cm^2^/Vs, −3.5 V, 0.75 V/decade and 8 × 10^5^, respectively. Due to the hysteresis, the threshold voltage was determined by only taking the graph in the forward direction into account. The field-effect mobility and threshold voltage are comparable to the values of spin-coated devices, and also, a high reproducibility was achieved, which is illustrated in Figure 10 by plotting 12 transfer curves of the µ-dispensed FS111 on SU-8 dielectrics showing, again, high uniformity. Moreover, the on/off-current ratio increased about more than one order of magnitude, and also, the subthreshold slope improved, due to the patterned active area [28]. In addition, the current ratio I_D_/I_G_ is now more than 10^5^, as shown in Figure S3c. One drawback, as seen in the characteristics, is the increased hysteresis, which probably arises due to an unoptimized drying process of the semiconductor. A typical indication is the “coffee-stain” rings seen in Figure 8, which are formed because of the outward convective flow of the solvent during the drying process, leading to an increased material transport of the semiconductor to the edges of the droplet. Profilometer measurements as depicted in Figure 11 showed a mean thickness of approximately 80 nm in the center regions of the droplet, but an increased thickness of around 300 nm of the “coffee-stain” rings. This effect could be avoided by using mixed-solvent systems in which a flow with a direction counter to that of the convective flow can be induced, the so-called Marangoni flow. Indeed, it has been shown with TIPS-PEN devices that the “coffee-stain” effect can be avoided, accompanied by a reduction of the hysteresis by using a mixed-solvent system, due to the better ordering of the crystals [29]. Interestingly, it should be noted that the hysteresis loop direction of spin-coated OFETs using FS111 on SU-8 is different from the one of the µ-dispensed devices with the same materials and architecture. Hysteresis effects showing a higher drain current in the transfer characteristic during back sweeping (on to off) than in the forward sweeping direction (off to on), as seen in the spin-coated transistors based on SU-8 and PVP, are typically caused by mobile ions in the dielectric or by (ferroelectric) polarization of the dielectric [30]. Such effects have been already observed for PVP dielectrics and were mainly ascribed to an insufficient cross-link reaction, leaving residual OH groups inside the layer, causing the slow polarization [13,31]. Similarly, the SU-8 film might also exhibit additional chemical species that are capable of slow polarization. However, the extent in both cases in this work is very limited, since only slight hystereses are visible. On the contrary, the oppositely directed hysteresis loop of µ-dispensed OFET devices indicates charge traps at the semiconductor/dielectric interface originating from dielectric surface functionalities, adsorbed small molecules at the interface or structural defects of the semiconductor [32]. In particular, the latter has been already identified to be a critical factor with our µ-dispensed devices, due to the “coffee-stain” effect and, thus, supports our suggestion that an optimized dispensing process leading to an improved ordering of the molecules would probably reduce hysteresis issues.

### 3.4. Long-Term Stability of µ-Dispensed Organic Transistors on Flexible Substrates

**Figure 12:**
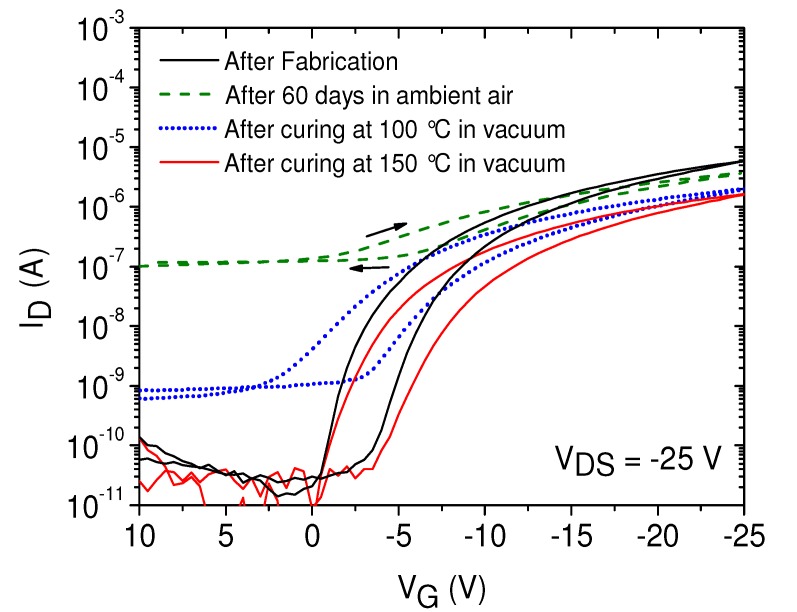
Transfer curves of organic transistors based on automated dispensing of FS111 on SU-8 dielectrics on PEN foil directly after fabrication and after 60 days using different curing temperatures.

In order to evaluate the long-term stability of the LCP-based transistors with SU-8 dielectrics, the devices exhibiting µ-dispensed FS111 were measured and characterized again after 60 days. The FOCs were stored in ambient air in light-transmissive wafer boxes under normal laboratory conditions (20 °C/40% relative humidity) and normal illumination (no amber light). A realistic storage condition in ambient atmosphere was chosen in order to identify different types of degradation. The transistors were measured without any prior treatment in the dark using the same measurement setup. A direct comparison of a transistor device measured as fabricated and after 60 days is plotted in Figure 12. The diagram shows that the off-current drastically increased about more than three orders of magnitude, and the on-current slightly decreased, reducing the on/off-ratio to a value of 40, accompanied by an increase of the subthreshold swing and threshold voltage. However, the charge carrier mobility remained almost the same after 60 days, which is promising stability data. We assumed a strong absorbance of water and oxygen molecules from the ambient air during the storage time, acting as additional mobile charges and, thus, shifting the off-currents to higher values. Therefore, a curing step in a vacuum oven at 100 °C for 10 min was carried out to remove any adsorbates in the polymers, leading to a decrease of the off-current of about two orders of magnitude. Although the on-current was also slightly decreased, the on/off-current ratio was improved to 3 × 10^3^. By using a higher curing temperature of 150 °C, we were able to further reduce the off-currents to the initial level, verifying a reversible degradation process of the semiconductor during the long-term ambient exposure. The mean values for the on/off ratio and field-effect mobility for these devices were 4 × 10^5^ and 0.05 cm^2^/Vs, respectively, indicating only a slight degradation of the FS111 after the exposure to ambient air for 60 days. Subthreshold swing and threshold voltage also recovered the initial values. Thus, even though the organic semiconductor was exposed to humidity and oxygen, irreversible degradation of the semiconductor is very limited, which is encouraging in regards to the transistor stability. The hysteresis seen in the initial characteristic was still present, and the threshold voltage was determined to be at −4 V in the graph in the forward direction; nevertheless, the transistors showed still good switching behavior. Table 2 summarizes the results for the automated µ-dispensed organic transistors with SU-8 dielectrics on foil.

**Table 2:** Extracted parameters for µ-dispensed FS111 using SU-8 dielectrics on FOC as fabricated and after 60 days with and without curing at elevated temperatures in a vacuum.

Characteristic	As fabricated	After 60 days	After 60 days and curing at 100 °C in a vacuum	After 60 days and curing at 150 °C in a vacuum
Field-effect mobility µ_sat_	0.09 cm^2^/Vs	0.07 cm^2^/Vs	0.05 cm^2^/Vs	0.05 cm^2^/Vs
Threshold voltage V_T_	−3.5 V	3 V	0 V	−4 V
On/off-current ratio	8 × 10^5^	50	3 × 10^3^	4 × 10^5^
Subthreshold swing	0.75 V/dec	80 V/dec	5 V/dec	0.8 V/dec

## 4. Conclusions

In summary, we evaluated the transistor performance based on a semiconducting liquid-crystal polymer using SU-8 and PVP dielectrics. We showed that SU-8 is a suitable dielectric material for OFET devices, which allows reducing the process temperature down to 100 °C, thus being used on low-temperature organic substrates, while maintaining the transistor performance, as compared to thermally-cured PVP on glass. The formation of a liquid-crystalline mesophase of the LCP material as proven by XRD measurements supports the realization of organic transistors with reliable device-to-device reproducibility on both dielectrics. Furthermore, we investigated the feasibility of an automated µ-dispensing process to improve the transistor performance and analyzed the long-term stability of the devices. The experiments were carried out using a foil-on-carrier technology enabling the highest versatility and comparability.

## Supplementary Materials

Supplementary materials can be accessed at: http://www.mdpi.com/1996-1944/7/11/7226/s1
