# Supplementary material for: Organic Field-Effect Transistors Based on a Liquid-Crystalline Polymeric Semiconductor using SU-8 Gate Dielectrics on Flexible Substrates
Source: Materials (Basel). 2014 Oct 29;7(11):7226–42. doi: 10.3390/ma7117226 (PMC5512632; doi:10.3390/ma7117226)

Supporting Information

Figure S1. Wide-angle X-ray diffraction spectra of FS111 as-cast and after annealing at 140 °C for 5 min.

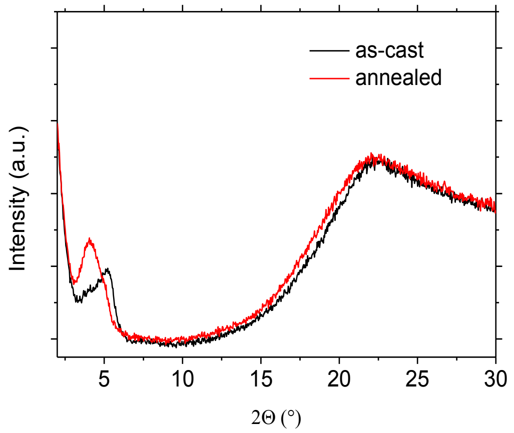

Figure S2. Transfer curve of a PVP-based OFET device using FS111 operating at −25 V with root square of I_D for parameter extraction (**a**) and gate leakage current for calculation of I_D/I_G-ratio (**b**).

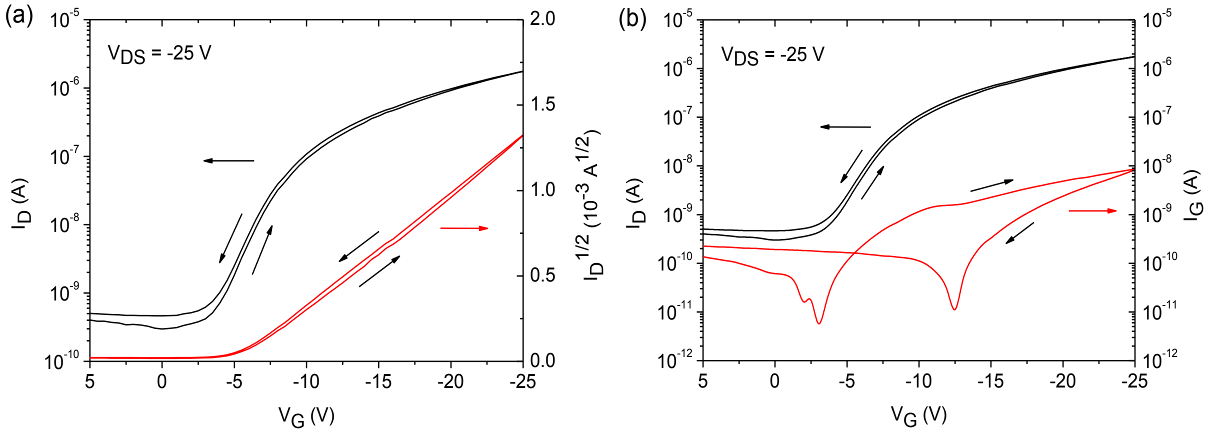

Table S1. Extracted parameters for FS111 using PVP dielectrics on glass at supply voltages of −25 V.

Characteristic	FS111 on PVP/glass at supply voltages of −25 V
Field-effect mobility μ_{sat}	0.1 cm²/Vs
Threshold voltage V_T	−5 V
On/Off-current ratio	5×10^3
Subthreshold Swing	2.6 V/dec

Figure S3. Transfer characteristics of (**a**) spin-coated FS111 on SU-8; (**b**) spin-coated FS111 on PVP and (**c**) automatically µ-dispensed FS111 on SU-8 demonstrating the relation of the drain current I_D to the gate current I_G.

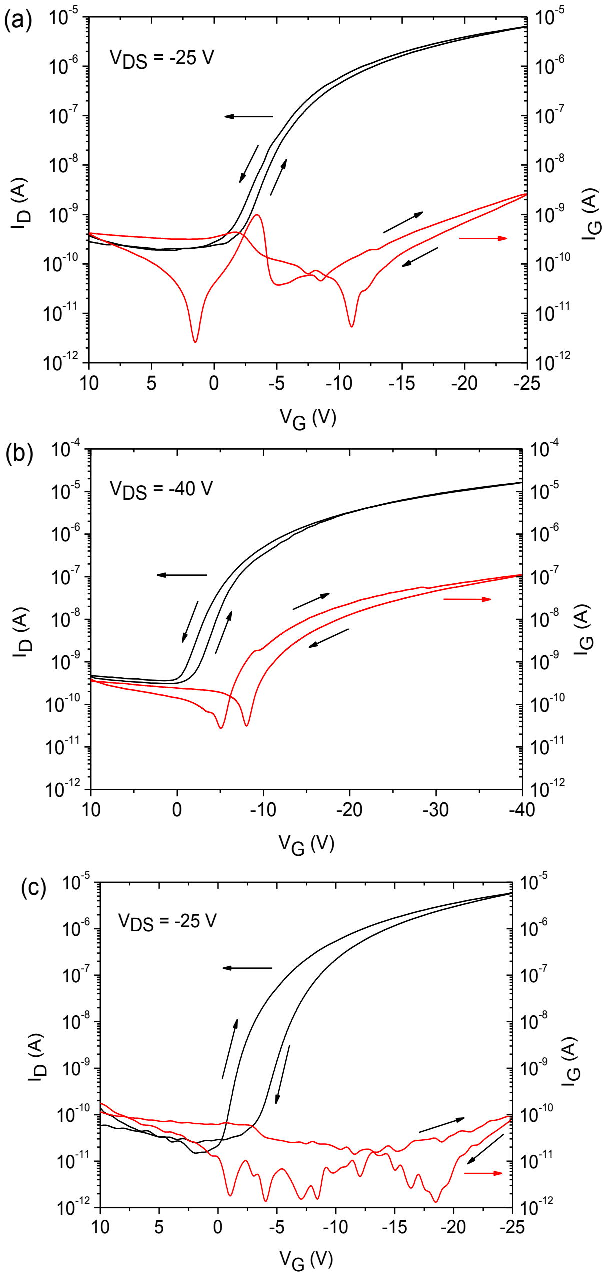

Supplement: Supplementary file 1 [file materials-07-07226-s001.pdf]
